# The effect of holes in long-lasting insecticidal nets on malaria in Malawi: results from a case–control study

**DOI:** 10.1186/s12936-017-2033-3

**Published:** 2017-10-02

**Authors:** Anna A. Minta, Keren Z. Landman, Dyson A. Mwandama, Monica P. Shah, Jodi L. Vanden Eng, James F. Sutcliffe, Joseph Chisaka, Kim A. Lindblade, Don P. Mathanga, Laura C. Steinhardt

**Affiliations:** 1 Malaria Branch, Division of Parasitic Diseases and Malaria, Centers for Disease Control and Prevention, 1600 Clifton Road, Atlanta, GA 30329 USA; 2 Epidemic Intelligence Service, Centers for Disease Control and Prevention, 1600 Clifton Road, Atlanta, GA 30329 USA; 3 Malaria Alert Centre, University of Malawi College of Medicine, Private Bag 360, Blantyre, Malawi

**Keywords:** Malaria, Physical integrity, Holes, Bed nets, Long-lasting insecticidal nets, Malawi

## Abstract

**Background:**

Long-lasting insecticidal nets (LLINs) are a cornerstone of malaria prevention. Holes develop in LLINs over time and compromise their physical integrity, but how holes affect malaria transmission risk is not well known.

**Methods:**

After a nationwide mass LLIN distribution in July 2012, a study was conducted to assess the relationship between LLIN damage and malaria. From March to September 2013, febrile children ages 6–59 months who consistently slept under LLINs (every night for 2 weeks before illness onset) were enrolled in a case–control study at Machinga District Hospital outpatient department. Cases were positive for *Plasmodium falciparum* asexual parasites by microscopy while controls were negative. Digital photographs of participants’ LLINs were analysed using an image-processing programme to measure holes. Total hole area was classified by quartiles and according to the World Health Organization’s proportionate hole index (pHI) cut-offs [< 79 cm^2^ (good), 80–789 cm^2^ (damaged), and > 790 cm^2^ (too torn)]. Number of holes by location and size, and total hole area, were compared between case and control LLINs using non-parametric analyses and logistic regression.

**Results:**

Of 248 LLINs analysed, 97 (39%) were from cases. Overall, 86% of LLINs had at least one hole. The median number of holes of any size was 9 [interquartile range (IQR) 3, 22], and most holes were located in the lower halves of the nets [median 7 (IQR 2, 16)]. There were no differences in number or location of holes between LLINs used by cases and controls. The median total hole area was 10 cm^2^ (IQR 2, 125) for control LLINs and 8 cm^2^ (IQR 2, 47) for case LLINs (*p* = 0.10). Based on pHI, 109 (72%) control LLINs and 83 (86%) case LLINs were in “good” condition. Multivariable modeling showed no association between total hole area and malaria, controlling for child age, caregiver education, and iron versus thatched roof houses.

**Conclusions:**

LLIN holes were not associated with increased odds of malaria in this study. However, most of the LLINs were in relatively good condition 1 year after distribution. Future studies should examine associations between LLIN holes and malaria risk with more damaged nets.

**Electronic supplementary material:**

The online version of this article (doi:10.1186/s12936-017-2033-3) contains supplementary material, which is available to authorized users.

## Background

Despite major improvements in control efforts, malaria remains a significant cause of morbidity and mortality worldwide, causing an estimated 212 million cases and 429,000 deaths in 2015 [1]. Insecticide-treated mosquito nets (ITNs) remain one of the most important tools for malaria prevention. The proportion of the at-risk population in sub-Saharan Africa sleeping under an ITN increased from 5% in 2000 to 53% in 2015, and models estimate that ITNs have contributed to reducing parasite prevalence among children 2–10 years old in sub-Saharan Africa by 50% between 2000 and 2015 [1]. The World Health Organization (WHO) recommends that ITNs used to prevent malaria be long-lasting insecticidal nets (LLINs), which do not have to be re-treated with insecticide [2]. In this manuscript, the term “bed net” will be used as a generic term to describe untreated nets, ITNs, or LLINs.

To maintain gains in malaria control from the introduction and wide-scale distribution of LLINs, while maintaining lowest possible costs, decisions on replacement frequency should be informed by evaluations of the impact of holes on the effective lifespan of LLINs [3]. The WHO and Vector Control Technical Expert Group (VCTEG) guidelines describe methods to assess LLIN durability based on net attrition, insecticide content, insecticidal activity, and physical integrity [3, 4]. Physical integrity is assessed using a proportionate Hole Index (pHI), a composite measure which is a weighted sum of the number of holes in each of four hole size categories. In the field, LLINs are removed from the home and draped over a frame for evaluation. Hole sizes are typically estimated based on their approximate diameter in relation to a thumb (0.5–2 cm), fist (2–10 cm), head (10–25 cm), and larger than a head (≥ 25 cm). Holes < 0.5 cm in diameter are not included in the assessment [3]. In order to make pHI comparable between studies, the WHO VCTEG equated the pHI with approximate total hole surface areas. Assuming holes are circular, LLINs are classified based on the following total holes areas: < 79 cm^2^ (“good”), 80–789 cm^2^ (“damaged”), and > 790 cm^2^ (“too torn”). Various methods, including WHO pHI, have been used in field studies to assess the loss of physical integrity in LLINs over time [5–8]. The length of time it takes an LLIN to develop holes depends on several factors including the LLIN brand, the age and gender of the person sleeping under the net, and household factors such as presence of rats and use of the bed net with a reed mat [6, 9–13].

To begin to understand the association between LLINs holes and malaria risk, studies have been done to assess how mosquitoes interact with nets. Laboratory and semi-field studies using human-baited, intact bed nets have shown that mosquitoes are more likely to attempt to enter either untreated or insecticide-treated bed nets through the roof than through the sides [14, 15]. Therefore, documentation of hole location as part of bed net integrity evaluations has been recommended [14, 15]. A systematic review of experimental hut studies describing holes in bed nets (untreated, ITNs, and LLINs) found that overall, mosquito feeding increased as holes in bed nets increased, but mosquito mortality was not affected by holes [16]. Two experimental hut studies not included in the systematic review have shown that the presence of effective insecticide may or may not mitigate mosquito biting through LLINs with holes [17, 18].

Only a few studies have assessed the association between physical integrity of bed nets and malaria risk in natural (i.e. non-laboratory or experimental hut) settings. A study using cross-sectional household survey data from Malawi, and from both Bioko Island and mainland Equatorial Guinea [all settings with low bed net use (30–39%)], found that children who reportedly slept under intact LLINs had lower odds of *Plasmodium falciparum* infection than children who slept under LLINs with holes, and that LLINs with “large” holes (≥ D sized torch battery) provided less protection than LLINs with “small” holes (< D sized torch battery) [19]. In another study using WHO criteria for measuring LLIN physical integrity, authors found a higher prevalence of positive malaria blood smears (23%) among households with a “moderately torn” or “too torn” net compared to a prevalence of 5% in households with a “good” net [9]. One case–control study found no difference in the odds of rapid diagnostic test (RDT)-diagnosed malaria among hospitalized children compared to healthy controls based on whether their ITN was in good condition or not (using a Likert scale) when examined during a household visit [20]. Some of the limitations to previous studies that examined the association between physical integrity and malaria include: use of parasite prevalence instead of clinical malaria as the outcome, use of subjective instead of objective measures of physical integrity, or categorization of holes in a way that cannot be easily replicated instead of systematically measuring holes. These shortcomings make it difficult to ascertain an association between laboratory-confirmed malaria and systematically measured holes in nets. In addition, field-based hole assessments typically provide a somewhat crude estimate of the total hole area, and the thumb/fist/head-sized categories used to determine WHO pHI can over- or underestimate various hole sizes [21, 22]. WHO also recommends the documentation of repaired holes when assessing physical integrity [4]; although, there is currently no evidence to suggest that repaired holes improve bed net condition [11, 13, 23, 24]. Finally, these studies did not systematically assess housing characteristics, such as roof or wall type, presence of screening or glass in windows, or open or closed eaves. Housing characteristics should be included in bed net studies because modern materials (compared to traditional materials) have been associated with protection from malaria [25].

The objectives of this study were to describe the number, location, and total hole area in LLINs, using digital image analysis, 1 year after a distribution campaign in Malawi and to assess the relationship between observed LLIN holes and laboratory-confirmed malaria infection among recently febrile pediatric outpatients who slept under the LLINs.

## Methods

### Study setting

This study was conducted at the Machinga District Hospital in southern Malawi. Malaria transmission in Malawi is year-round, with a peak during the November–May rainy season. The primary vector for malaria transmission is *Anopheles funestus* [26]. A national mass distribution of Olyset^®^ Net (Sumitomo Chemical Co., Japan) LLINs was completed in July 2012, with one LLIN distributed for every two people. Indoor residual spraying has not been conducted in this area.

### Study design

From March to September 2013, children 6–59 months who presented to the under-five outpatient clinic at Machinga District Hospital were recruited into a case–control study to assess the personal protective effect of LLINs on malaria morbidity [27]. Inclusion criteria were residence within 15 km of the hospital and axillary temperature ≥ 37.5 °C (as measured by the survey team) or history of fever within the past 48 h (per caregiver report). After written informed consent was obtained from the caregiver, participants were enrolled in an overall study of the association between bed net use and malaria risk. Surveyors administered a questionnaire that included information on illness history, socioeconomic status, malaria risk factors, and LLIN use. Thick and thin blood films as well as an RDT for malaria (Paracheck Pf^®^ device, Orchid Biomedical Systems, India) were obtained. The microscopists were blinded to the RDT results. All children with a positive RDT were treated by staff at the health facility according to national guidelines. Cases were defined as children positive for *P. falciparum* asexual parasitemia by microscopy, and controls were negative for *P. falciparum*. Children whose caregivers reported that their ill child had slept under an LLIN obtained from the recent campaign for 14 of 14 nights prior to illness onset were eligible for this sub-study on LLIN physical integrity.

### Home visit and LLIN analysis

Within 2 weeks of the clinic visit, surveyors conducted home visits, during which they assessed housing characteristics and visually inspected the home’s exterior environment for potential mosquito breeding sites within 20 m of the home. The caregiver identified the child’s LLIN, and surveyors exchanged the child’s LLIN with a new one. Participants’ LLINs were brought to the study clinic and mounted on a metal frame with a black cloth draped over it. Surveyors assessed the size and location of holes on each side and the roof of the LLIN based on the standard WHO protocol [4]. A Nikon Digital Coolpix Camera (model number AW100V1.0, 16 Megapixels, Nikon Corporation, Tokyo, Japan) was used to take digital photographs of the roof, upper half of each side, and lower half of each side of the LLIN. The photographs had 4608 × 3456 resolution and were sent to the United States-based Centers for Disease Control and Prevention (CDC) as JPEG files for image analysis.

### Hole analysis

An image processing program called ImageJ (IJ 1.46r, National Institutes of Health, Rockville, Maryland) was used to determine exact hole measurements. Each hole was highlighted by hand, and the area (cm^2^) of each hole was calculated by the program. Additional measurements were calculated, such as aspect ratio and circularity, which are described in detail by Vanden Eng JL et al. (pers. comm.).

### Data analysis

Descriptive statistics were calculated for participant demographics and household characteristics assessed by the home visit. Child’s age was analysed as a continuous variable and as a categorical variable (6–12 and 13–59 months). A socioeconomic status (SES) index was calculated using principal component analysis on 12 key household factors including: electricity, caregiver’s and spouse’s occupation, ownership of various assets, source of water, and type of toilet [28]. The SES index was divided into quintiles. Household characteristics (open vs closed house eaves, presence of windows, roof material, and wall material) were excluded from the SES index, and collinearity between household characteristics was assessed using Spearman’s correlation coefficient.

LLIN physical integrity was characterized in the following ways: total number of holes in the LLIN, the number of holes of various sizes, the number of holes by location, and total hole area. Holes ≥ 10 cm diameter include the two largest holes sizes by WHO criteria. LLINs were categorized based on total hole areas into the following categories: < 79 cm^2^ (“good”), 80–789 cm^2^ (“damaged”), and > 790 cm^2^ (“too torn”), as suggested by the WHO VCTEG, as well as quartiles.

Comparisons between case and control children were made using the Chi square statistic (Fischer’s exact test was used for expected counts < five) for binary and categorical variables, or the Wilcoxon rank-sum test for continuous variables. Logistic regression analyses were conducted with malaria as the outcome and LLIN physical integrity as the primary exposure. Potential confounders (demographics and housing characteristics) were included in multivariable model building. The final multivariable model included variables in the model with the lowest Akaike information criterion (AIC) [29].

### Ethics approval and consent to participate

Written informed consent was obtained from parents or caregivers of each study participant prior to enrollment in the study. The study protocol was reviewed and approved by the institutional review boards of the University of Malawi, College of Medicine, Blantyre, Malawi and the Centers for Disease Control and Prevention, Atlanta, GA, USA (Protocol Number 6414).

## Results

The main case–control study included 1181 participants [27]. There were 426 children whose caregiver reported during the clinic visit that the child slept under an LLIN for 14 nights prior to illness onset and used an Olyset^®^ LLIN distributed during the mass campaign, and thus were eligible for the sub-study on net integrity. Some homes could not be located or the caregiver declined the home visit, so 320 children (75%) had a home visit. During the home visit, 276 (86%) of the caregivers agreed to exchange the child’s LLIN for the assessment. When the field staff assessed the nets, some nets were not Olyset^®^ brand and these were excluded, which left 248 (90%) participants with questionnaire, home visit, and Olyset^®^ LLIN image analysis data who were included in the current study.

Of the 248 participants, 97 (39%) had laboratory-confirmed malaria (Table 1). The proportion of cases in the original case–control study was similar (34%, p = 0.13, Chi square). The median age of participants was 25 months [interquartile range (IQR) 16, 37], and approximately half were female. The median age of caregivers was 26 years (IQR 22, 31), and 181 (73%) had a primary school education. Most children slept on the floor or on a mat (n = 198, 80%) versus on a mattress. The median number of nets owned per household was 2 (IQR 1, 2), with a median of 1 (IQR 1, 2) reported to be hanging. Very few participants (n = 12, 5%) used any other mosquito repellant measure, such as mosquito coils or mosquito spray. Home visits revealed that 55 (22%) households had identified potential mosquito breeding sites, such as puddles and open drains, within 20 m of the home. In most homes, some or all of the eaves were open (n = 201, 81%) compared to all closed. Most homes had windows (n = 181, 73%), but very few of these homes had screens on all windows (n = 4, 2%). Most homes were constructed with thatch or palm leaf roofs (n = 179, 73%) and cement or brick walls (n = 209, 84%).

**Table 1:** Demographics and housing characteristics for enrolled children with recent fever presenting to outpatient department, (n = 248)

Variable	Total (n= 248)	Control children (n = 151)	Case children (n=97)	p value
Assessed by parental report				
	N (%)	N (%)	N (%)	
Age categories, months				
6–12	47 (19.0)	35 (23.2)	12 (12.4)	
13–59	201 (81.1)	116 (76.8)	85 (87.6)	0.03*
Sex				
Male	123 (49.6)	74 (49.0)	49 (50.5)	
Female	125 (50.4)	77 (51.0)	48 (49.5)	0.82
Education of caregiver				
None	25 (10.1)	10 (6.7)	15 (15.5)	
Primary	181 (73.3)	110 (73.3)	71 (73.2)	
Secondary and higher	41 (16.6)	30 (20.0)	11 (11.3)	0.03*
Caregiver age (years), median (IQR)	26 (22, 31)	25 (22, 31)	28 (23, 32)	0.22
Socio-economic status				
Lower 80%	196 (79.4)	117 (78.0)	79 (81.4)	
Upper 20%	51 (20.7)	33 (22.0)	18 (18.6)	0.51
Child sleeps on				
Mattress	50 (20.2)	36 (23.8)	14 (14.4)	
Floor/mat	198 (79.8)	115 (76.2)	83 (85.6)	0.07
Number of nets owned, median (IQR)	2 (1, 2)	2 (1, 2)	2 (1, 2)	0.30
Number of nets hanging, median (IQR)	1 (1, 2)	1 (1, 2)	1 (1, 2)	0.49
Household use of any mosquito repellant measures^a^				
Yes	12 (4.8)	7 (4.6)	5 (5.2)	1.00
Flooring type				
Non-cement	206 (83.4)	119 (79.3)	87 (89.7)	
Cement	41 (16.6)	31 (20.7)	10 (10.3)	0.03*
Assessed at home visit				
Breeding sites within 20 meters^b^				
Yes	55 (22.2)	34 (22.5)	21 (21.7)	0.87
House eaves				
All or some open	201 (81.1)	116 (76.8)	85 (87.6)	
All closed	47 (19.0)	35 (23.2)	12 (12.4)	0.03*
Windows				
No windows	66 (26.6)	40 (26.5)	26 (26.8)	
Windows, not screened or some screened	178 (71.8)	109 (72.2)	69 (71.1)	
Windows, all screened	4 (1.1)	2 (1.3)	2 (2.1)	0.92
Presence of at least 2 windows on different walls				
Yes	71 (28.6)	49 (32.5)	22 (22.7)	0.10
Number of nets hanging, median (IQR)	1 (1, 2)	1 (1, 2)	1 (1, 1)	0.44
Roof type^c^				
Thatch/palm leaf	179 (72.8)	98 (65.3)	81 (84.4)	
Non-thatch/palm leaf	67 (27.2)	52 (34.7)	15 (15.6)	0.001*
Exterior wall material^d^				
Natural materials	39 (15.7)	24 (15.9)	15 (15.5)	
Cement or brick	209 (84.3)	127 (84.1)	82 (84.5)	0.93

* p value < 0.05

^a^Mosquito repellant, mosquito coils, or spraying with insecticide

^b^Examples of potential breeding sites include puddles, swamps, and open drains, among others

^c^Non-thatch/palm leaf includes calamine/cement fiber, ceramic tiles, or corrugated galvanized iron sheets

^d^Natural materials include cane, palm, trunks, or dirt walls. Cement or brick includes cement and burnt or unburnt brick walls

Case children were more likely to be older (13–59 months) than control children (88% vs 77%, p = 0.03) and were more likely to have caregivers with no formal education (16% vs 7%, p = 0.03, see Table 1). Case children were more likely than control children to live in homes with thatched or palm roofs (84% vs 65%, p = 0.001).

Among assessed LLINs, 223 (90%) had at least one hole of any size and 214 (86%) had at least one hole > 0.5 cm in diameter (Table 2). Forty-three LLINs (17%) had at least one hole ≥ 10 cm diameter. The median number of holes of any size was 9 (IQR 3, 22) for all nets, 9 (IQR 4, 20) for case LLINs, and 9 (IQR 2, 23) for control LLINs. Most holes were located on the lower halves of LLINs, and there was no difference between case LLINs [median number of holes in the lower half = 6, (IQR 2, 13)] and control LLINs [median = 7, (IQR 1, 18)]. The median number of holes in the upper halves of LLINs was two for both case LLINs (IQR 0, 5) and control LLINs (IQR 0, 6). For both case and control LLINs, nearly all LLINs had no holes in the roof [median = 0, (IQR 0, 2)]. The median number of repaired holes for all LLINs, cases, and controls was 0 (IQR 0, 0) and 47 LLINs (19%) had at least one repaired hole. The proportion of LLINs with at least one repaired hole between case LLINs and controls nets was similar (p = 0.62).

**Table 2:** LLIN characteristics for case and control children

Bed Net Parameters	Total, n = 248	Control children, n = 151	Case children, n = 97	p value
	N (%) or Median (IQR)	N (%) or Median (IQR)	N (%) or Median (IQR)	
Nets with at least one hole > 0.5 cm diameter	214 (86.3)	127 (84.1)	87 (89.7)	0.26
Nets with at least one hole of any size	223 (89.9)	133 (88.1)	90 (92.8)	0.28
Nets with at least one hole ≥ 10 cm diameter	43 (17.3)	30 (19.9)	13 (13.4)	0.23
Total number of holes of any size	9 (3, 22)	9 (2, 23)	9 (4, 20)	0.82
Total number of holes > 0.5 cm diameter	7 (2, 18)	7 (2, 19)	6 (3, 14)	0.91
Number holes in net roof	0 (0, 2)	0 (0, 2)	0 (0, 2)	0.41
Number of holes in upper half of net	2 (0, 6)	2 (0, 6)	2 (0, 5)	0.68
Number of holes lower half of net	7 (2, 16)	7 (1, 18)	6 (2, 13)	0.63
Repaired holes				
None	201 (81.1)	124 (82.1)	77 (79.4)	
≥ 1	47 (19.0)	27 (17.9)	20 (20.6)	0.62
Total hole area (cm^2^)	9.4 (1.8, 69.8)	9.9 (1.7, 124.6)	8.3 (2.2, 47.3)	0.52
pHI				
Good (< 79 cm^2^)	192 (77.4)	109 (72.2)	83 (85.6)	
Damaged (80–789 cm^2^)	40 (16.1)	30 (19.9)	10 (10.3)	
Too torn (> 790 cm^2^)	16 (6.5)	12 (8.0)	4 (4.1)	0.048*
Total hole area quartiles				
Q1: (≤ 1.8 cm^2^)	62 (25.0)	40 (26.5)	22 (22.7)	
Q2: (> 1.8–≤ 9.5 cm^2^)	62 (25.0)	34 (22.5)	28 (28.9)	
Q3: (9.5–≤ 70.0 cm^2^)	62 (25.0)	33 (21.9)	29 (29.9)	
Q4: (> 70.0–≤ 2561.6 cm^2^)	62 (25.0)	44 (29.1)	18 (18.6)	0.14

* p value < 0.05

**Fig. 1:**
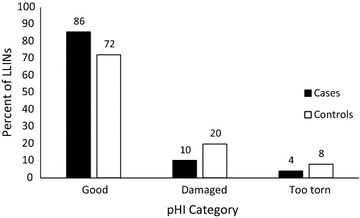
Percent of LLINs Used by Participants by pHI Category

Median total hole area was 10 cm^2^ (IQR 1.7, 124.6) for control LLINs and 8.3 cm^2^ (IQR 2.2, 47.3) for case LLINs (p = 0.52). When the total hole area was divided into pHI serviceability categories, most LLINs were in “good” condition [n = 192 (77%)], followed by “damaged” [n = 40 (16%)] and “too torn” [n = 16 (7%)]. LLINs from cases tended to be in somewhat better condition compared to LLINs from controls, according to the WHO pHI categories, p = 0.048; however, total hole area, when divided into quartiles, was not significantly different between the two groups (Fig. 1 and Table 2).

In univariable logistic regression modeling, LLINs in “good” vs “damaged” pHI category (p = 0.04), older age of the child (p = 0.04), lower caregiver education (p = 0.01), open house eaves (p = 0.04), and living in a house with a thatch/palm roof (p = 0.001) were associated with higher odds of malaria (Table 3). No differences between case and control children were found for SES, number of nets found hanging, presence of nearby potential breeding sites, presence of windows on opposite walls, or exterior wall material. There was no association between total hole area, divided into quartiles, and malaria (Additional file 1: Table S1). Of note, roof type was collinear with open vs closed eaves (Spearman’s correlation coefficient = 0.70). The most parsimonious multivariable model, using Akaike information criterion, for the association between pHI and malaria included child’s age, caregiver’s age, and roof material. Adjusted for these confounders, the association between pHI and malaria was no longer significant (p = 0.07). In the final multivariable model, older age of the child (p = 0.04) and living in a house with thatch/palm leaf roof (p = 0.002) were associated with higher odds of malaria.

**Table 3:** Univariable and multivariable logistic regression analysis of malaria

Variable	Univariable analysis^a^			Multivariable analysis^b^		
	Unadjusted odds ratio (OR)	95% confidence interval (CI)	p value	Adjusted OR	95% CI	p value
pHI category						
Good (< 79 cm^2^)	Reference			Reference		
Damaged: (80–789 cm^2^) vs good	0.44	[0.20–0.95]	0.04*	0.48	[0.21–1.06]	0.07
Too torn (> 790 cm^2^) vs good	0.44	[0.14–1.41]	0.17	0.46	[0.14–1.52]	0.20
Age categories, months						
6–12	Reference			Reference		
13–59	2.14	[1.05–4.36]	0.04*	2.15	[1.02–4.53]	0.04*
Education of caregiver						
None	Reference			–	–	–
Primary	0.43	[0.18–1.01]	0.05	–	–	–
Secondary and higher	0.24	[0.09–0.70]	0.01*	–	–	–
Caregiver age	1.03	[0.99–1.07]	0.16	1.03	[0.99–1.07]	0.15
SES index						
Lower 80%	Reference					
Upper 20%	0.81	[0.43–1.53]	0.51	–	–	–
Child sleeps on						
Floor/mat	Reference					
Mattress	0.54	[0.27–1.06]	0.07	–	–	–
Potential breeding sites						
No	Reference					
Yes	0.95	[0.51–1.76]	0.87	–	–	–
House eaves						
All or some open	Reference					
All closed	0.47	[0.23–0.95]	0.04*	–	–	–
At least 2 windows on different walls						
No	Reference					
Yes	0.61	[0.34–1.10]	0.10	–	–	–
Number of nets hanging	0.89	[0.61–1.29]	0.52	–	–	–
Roof type						
Thatch/palm leaf	Reference			Reference		
Non-thatch/palm leaf	0.35	[0.18–0.67]	0.001*	0.34	(0.17-0.66)	0.002*

* p value < 0.05

^a^Results of odds ratios for sex, use of repellant, wall materials, and presence of screened windows are not included in the table because they were similar between cases and controls

^b^All variables in Table 1 were included in multivariable model building. The final multivariable model presented is the model with lowest AIC

## Discussion

In this study, there was not a significant association between confirmed malaria and LLIN physical integrity, measured in terms of number, size, location, and total hole area. Holes were systematically assessed using digital image analysis that can capture the exact hole area, rather than relying on calculations and assumptions of circularity. Previous studies have examined the association between LLIN physical integrity and mosquito behavior or mosquito biting; however, the link between LLIN physical integrity and malaria cannot be determined from these studies. This paper presents epidemiological data to evaluate the association between LLIN damage and clinical malaria. This study is novel because cases had laboratory-confirmed, clinically patent malaria illness, all participants reported consistent LLIN use, and participants’ LLINs were photographed and analysed digitally for precise hole measurements. Digital image analysis may improve on standard methods using surveyor assessment by calculating the exact area of the hole, because field assessments can over- or underestimate the true hole area ([21] and Vanden Eng JL et al., pers. comm.).

More than three-quarters of the LLINs in this study were in “good” condition according to WHO criteria, which is reassuring 1 year after an LLIN campaign. The combination of insecticide in the LLINs and overall “good” physical integrity suggests that most of the evaluated LLINs should have been effective in providing protection for the individuals sleeping under them, and may help explain why there was no significant association between malaria and the physical integrity of the LLINs. Previous studies have shown that mosquito feeding is still inhibited by LLINs with a total hole area of 96 cm^2^ [16]. The median LLIN total hole area in this study was only 9.4 cm^2^, suggesting that the risk of malaria may not be affected by a small amount of LLIN damage. Laboratory studies have shown that mosquitoes are more likely to enter bed nets through the roof [14, 15], but the small number of roof holes in the LLINs in this study meant that the association between roof holes and malaria could not be assessed. The population in this study area has high LLIN usage; in fact, 86% of children in the larger case–control study reportedly used an LLIN the night before they were surveyed [27]. High ITN use in the community contributes to a community protective effect so that people who do not use ITNs are also protected from malaria [30]. The high LLIN use in the study area may have compensated for any loss in protection from the small total hole area seen in individual LLINs. This conclusion is supported by the larger case–control study that found no association between LLIN use and malaria at the individual level [27], and by another study that found no association between ITN integrity and malaria among hospitalized children in a setting where ITN ownership was > 80% [20]. The lack of significant association between LLIN physical integrity and malaria might be due to limited sample size, particularly the small number of moderate-to-severely damaged LLINs in the study sample.

In univariable analysis, the odds of malaria were significantly lower for children who slept under a “damaged” or “too torn” LLIN than a “good” LLIN. Although this result is no longer significant in multivariable analysis, and no significant relationship was found between total hole area categorized into quartiles and malaria, this finding is unexpected. The association, based on univariable analysis, is possibly related to the small number of “damaged” and “too torn” nets in this study or to residual confounding by factors that were not measured in the study.

This study highlights the association between housing material and construction (roof type and open vs closed eaves) and malaria among children. A recent meta-analysis described a consistent protective effect of “modern” materials and housing construction such as concrete walls, iron roofs, and closed eaves over traditional materials and construction such as mud floors and thatched roofs, presumably because traditional housing materials are more porous and provide more conducive places for mosquitoes to enter and rest in homes [25].

### Limitations

Reporting bias may be a limitation. At least one study has shown that caregiver-reported bed net use is higher in the clinic setting than in household surveys, the global standard for tracking bed net use [31]. If participants in this study did not actually use their LLIN consistently and this varied by case or control status, the results of the study could have been affected. Caregivers were asked about bed net use before results of their child’s malaria status were shared with them, so this is unlikely. In addition, a study conducted in the same part of Malawi as this study found high agreement between caregiver report that the child slept under an LLIN and the presence of hanging LLINs by household visits [32]. It is possible that when the LLINs were draped over the frame for evaluation, measurement error was introduced if the holes became stretched. Because the WHO recommends draping an LLIN over a frame for field evaluation, this potential measurement error should not have affected the comparison of the image analysis measurement with pHI cutoffs. Lastly, there is known moderate pyrethroid resistance near the study area (*Anopheles funestus*: 25% mortality with permethrin at 0.75% concentration and 38% mortality with deltamethrin at 0.05% concentration using the WHO tube assay method) [33], and insecticide content and insecticidal activity were not measured in this study. Presumably, both were still relatively high given that the nets were on average only one year old, and it is unlikely that the insecticide would differentially affect case and control LLINs.

## Conclusions

This study did not find any significant association between malaria and physical integrity of LLINs, measured by number and locations of holes, or total hole area categorized by pHI or into quartiles. LLINs in this study were in “good” condition, according to WHO pHI categories, which is reassuring 1 year after a mass campaign. Because the LLINs were in “good” condition and were insecticide treated, they were likely still effective in providing individual protection, and high LLIN use in the area provided community protection. Similar studies should be conducted using older nets (2–3 years old), or nets with more numerous and larger holes which might be more susceptible to increased mosquito entry and thus increased malaria risk. In addition, studies should be replicated in different populations whose LLINs may degrade faster than the LILNs in this study. As additional studies are needed to better understand the association between malaria and LLIN physical integrity, current field methods for evaluating physical integrity to determine LLIN replacement timelines should continue until other methods of measuring LLINs are validated.

## Additional file

**Additional file 1: Table S1.** Sensitivity analysis: Association between malaria and total hole area quartiles.

## Abbreviations

AIC: Akaike information criterion
CDC: Centers for Disease Control and Prevention
ITN: insecticide-treated mosquito net
LLIN: long-lasting insecticidal net
pHI: proportionate hole index
RDT: rapid diagnostic test
SES: socioeconomic status
VCTEG: Vector Control Technical Expert Group
WHO: World Health Organization
