# Supplementary material for: The effect of holes in long-lasting insecticidal nets on malaria in Malawi: results from a case–control study
Source: Malar J. 2017 Oct 2;16:394. doi: 10.1186/s12936-017-2033-3 (PMC5625742; doi:10.1186/s12936-017-2033-3)
Supplement: Supplementary file 1 — Additional file 1: Table S1. Sensitivity analysis: Association between malaria and total hole area quartiles. [file 12936_2017_2033_MOESM1_ESM.docx]

Table S1. Sensitivity analysis: Association between malaria and total hole area quartiles

| LLIN total hole area by quartiles | Unadjusted Odds Ratio (OR) | 95% Confidence Interval (CI) | P value |
| --- | --- | --- | --- |
| Q1: (≤1.8 cm^2^) | Reference |  |  |
| Q2: (>1.8 to ≤ 9.5 cm^2^) vs Q1 | 1.50 | [0.73–3.08] | 0.27 |
| Q3: (9.5 to ≤ 70.0 cm^2^) vs Q1 | 1.60 | [0.78–3.29] | 0.20 |
| Q4: (>70.0 to ≤ 2561.6 cm^2^) vs Q1 | 0.74 | [0.35–1.58] | 0.44 |
